# Modulation of Hydrogen Peroxide-Induced Oxidative Stress in Human Neuronal Cells by Thymoquinone-Rich Fraction and Thymoquinone via Transcriptomic Regulation of Antioxidant and Apoptotic Signaling Genes

**DOI:** 10.1155/2016/2528935

**Published:** 2015-12-28

**Authors:** Norsharina Ismail, Maznah Ismail, Nur Hanisah Azmi, Muhammad Firdaus Abu Bakar, Hamidon Basri, Maizaton Atmadini Abdullah

**Affiliations:** ^1^Nutricosmeceuticals and Nutrigenomics Programme, Laboratory of Molecular Biomedicine, Institute of Bioscience, Universiti Putra Malaysia (UPM), 43400 Serdang, Selangor, Malaysia; ^2^Department of Nutrition and Dietetics, Faculty of Medicine and Health Sciences, Universiti Putra Malaysia (UPM), 43400 Serdang, Selangor, Malaysia; ^3^Department of Medicine, Faculty of Medicine and Health Sciences, Universiti Putra Malaysia (UPM), 43400 Serdang, Selangor, Malaysia; ^4^Department of Pathology, Faculty of Medicine and Health Sciences, Universiti Putra Malaysia (UPM), 43400 Serdang, Selangor, Malaysia

## Abstract

*Nigella sativa* Linn. (*N. sativa*) and its bioactive constituent Thymoquinone (TQ) have demonstrated numerous pharmacological attributes. In the present study, the neuroprotective properties of Thymoquinone-rich fraction (TQRF) and TQ against hydrogen peroxide- (H_2_O_2_-) induced neurotoxicity in differentiated human SH-SY5Y cells were investigated. TQRF was extracted using supercritical fluid extraction while TQ was acquired commercially, and their effects on H_2_O_2_ were evaluated using cell viability assay, reactive oxygen species (ROS) assay, morphological observation, and multiplex gene expression. Both TQRF and TQ protected the cells against H_2_O_2_ by preserving the mitochondrial metabolic enzymes, reducing intracellular ROS levels, preserving morphological architecture, and modulating the expression of genes related to antioxidants (SOD1, SOD2, and catalase) and signaling genes (p53, AKT1, ERK1/2, p38 MAPK, JNK, and NF-*κβ*). In conclusion, the enhanced efficacy of TQRF over TQ was likely due to the synergism of multiple constituents in TQRF. The efficacy of TQRF was better than that of TQ alone when equal concentrations of TQ in TQRF were compared. In addition, TQRF also showed comparable effects to TQ when the same concentrations were tested. These findings provide further support for the use of TQRF as an alternative to combat oxidative stress insults in neurodegenerative diseases.

## 1. Introduction

The rapid progress in the understanding of the human genome has opened up new avenues for studying interactions between nutrients and the genome [1]. Up to now, it has been clearly shown that one common mechanism that mediates cellular longevity and healthful aging is protection against oxidative stress [2]. Notably, an excessively high level of reactive oxygen species (ROS) with normal levels of endogenous antioxidant enzymes is the basis for oxidative stress in the brain, which causes apoptosis and cell damage. In view of this, the use of exogenous antioxidants has been proposed as a method for managing ROS sequelae including damage to neuronal cells [3]. A major class of enzymatic antioxidants, which catalyze the dismutation of O_2_^−^ to H_2_O_2_, is known as superoxide dismutase (SOD). Multiple isoforms of SOD exist in different cellular compartments. SOD1 (CuZnSOD) is the major superoxide scavenger found in cytoplasm, mitochondrial intermembrane spaces, nuclei, and lysosomes, whereas SOD2 (MnSOD) is found in the mitochondria [4]. Further conversion of H_2_O_2_ to H_2_O + O_2_ occurs through the action of catalase, a heme-based enzyme which is normally localized in the peroxisome [5].

Besides manipulating ROS levels by using antioxidants, cell death and survival can also be modulated by targeting redox-sensitive signaling molecules at the signal transduction, transcription, or death-execution levels [6]. For instance, at the signal transduction level, mitogen-activated protein kinases (MAPK) are involved. Three major MAPK have been identified: ERK1/2, c-Jun N-terminal kinase (JNK), and p38 MAPK. JNK activation was found to be associated with ROS-induced neuronal death in Parkinson's and Alzheimer's disease. Chemical inhibitors of this signaling pathway have proven to be effective in vivo to reduce brain damage in animal models [7]. In addition, oxidative stress-induced activation of the PI3K/Akt pathway is crucial for cell survival [8]. At the transcription level, redox-sensitive transcription factors like NF-*κ*B regulate expression of multiple antioxidants. ROS can either activate or inhibit NF-*κ*B activity, depending on the level of ROS, types of stimuli, and cell types [9, 10]. A moderate increase in ROS often leads to NF-*κ*B activation, which requires sequential steps in the cytosol and nucleus. Conversely, a severe increase of ROS can inactivate NF-*κ*B, leading to cell death. At the execution level, p53 is partly involved. Because molecular pathways of apoptosis are excessively activated in aging and neurodegenerative disorders, pharmacologic and genetic inhibition of apoptotic players such as p53 are emerging strategies to prevent or retard degenerative diseases [11].

The appeal of natural products over synthetic pharmaceuticals has stemmed from the cost-effectiveness and fewer side effects of natural products. As such, products like* Nigella sativa* (*N. sativa*) have received heightened interest. An important challenge, however, is how to maximize benefits from such natural bioresources. This has led to the development of bioactive-rich fractions, which have high concentrations of a lead compound and lower concentrations of other bioactive compounds that have been demonstrated to synergistically improve the bioactivity of the fractions [12]. The lead compound in* N. sativa* has been identified as Thymoquinone (TQ). The yellow crystalline molecule TQ (2-methyl-5-isopropyl-1,4-benzoquinone) has a basic quinone structure consisting of a para-substituted dione conjugated to a benzene ring to which methyl and isopropyl side chain groups are attached. TQ was found to exert its biological function by modulating the physiological and biochemical processes involved in ROS generation. In normal tissue, TQ acts as an antioxidant, whereas in tumors TQ induces ROS generation. Thus, TQ has a dual role, depending on the cellular microenvironment; it may act as an antioxidant or a prooxidant [13].

As TQ is the lead constituent in* N. sativa* and has demonstrated tremendous pharmacological attributes, thus, the TQ-rich fraction (TQRF) was developed in our laboratory by supercritical fluid carbon dioxide extraction (SFE). As described in our previous study [14], SFE is preferable for the extraction of* N. sativa* seed oil in comparison with conventional solvent extraction and hydrodistillation methods because it uses carbon dioxide gas to extract the oil and is solvent-free, nontoxic, and environmentally friendly. The oil extraction is also carried out under low temperature and oxygen-free conditions, thus preserving the TQ compound, which is highly susceptible to oxidative degradation. In this study, the fixed oil of* N. sativa* was first extracted and trapped in the first collection vessel and the oil was subsequently fractionated into a second vessel to produce TQRF. This process was carried out by adjusting the SFE parameters (i.e., pressure and temperature) which could be controlled to enable the system to target and concentrate bioactive compounds in a short period of time. As a result, only TQRF comprising volatile oil was transferred to the second collection vessel, and the fixed oil was left in the first vessel. Thus, together with TQ, TQRF contains other essential oils of* N. sativa* such as p-cymene, *α*-thujene, *α*-pinene, camphene, sabinene, *β*-pinene, *β*-myrcene, *α*-phellandrene, limonene, *γ*-terpinene, terpinolene, camphor, carvone, thymol, carvacrol, longicyclene, and borneol [15]. To our knowledge, there has been limited research carried out on the mechanism of action underlying the protective effect of* N. sativa* or TQ on cellular oxidative stress and signaling parameters, particularly in neurodegenerative diseases. Ahmad et al. [16] suggested that further research should focus on and explore the specific cellular and molecular targets of various constituents of* N. sativa*, particularly TQ. In the case of TQRF, we found that many of its effects are mediated at the transcriptional level via regulation of several genes [12].

In this regard, the present study investigated the neuroprotective properties of SFE-derived TQRF and commercially acquired TQ against H_2_O_2_-induced oxidative stress in differentiated human neuroblastoma SH-SY5Y cells. Their neuroprotective properties were assessed by cell viability assay, ROS assay, and morphological observation. The nutrigenomic basis underlying the neuroprotective effects of TQRF and TQ was assessed from the transcriptional patterns of the antioxidants and apoptosis-related genes in the human neuronal cells using a multiplex genetic analysis system (GeXP).

## 2. Materials and Methods

### 2.1. Reagents

The human neuroblastoma SH-SY5Y cell line was purchased from ATCC (VA, USA). Dulbecco's Modified Essential Eagle's Medium-Ham's Nutrient Mixture F-12 (DMEM-F12), fetal bovine serum, gentamicin, and phosphate-buffered saline (PBS) were obtained from Sigma (St. Louis, MO, USA). Total RNA Isolation kit was purchased from RBC Bioscience Corp. (Taipei, Taiwan), while GenomeLab GeXP Start Kit was purchased from Beckman Coulter Inc. (Miami, FL, USA), and magnesium chloride (MgCl_2_) and DNA Taq polymerase were from Thermo Fisher Scientific (Pittsburgh, PA, USA).

### 2.2. Extraction of TQRF by SFE System

TQRF was prepared according to our previous study [14] using an SFE system (Thar 1000 F, Thar Technologies, Inc., Pittsburgh, PA, USA). Briefly, 100 g of ground* N. sativa* seeds was placed into the SFE vessel and extraction parameters were set at 600-bar pressure, temperature of 40°C, and a carbon dioxide flow rate of 30 g/min. TQRF was collected from the collection vessel when the pressure was in the range of 100–300 bar and temperature was 40–60°C, after completion of the extraction process.

### 2.3. Cell Culture

The SH-SY5Y cells were maintained in complete culture medium containing DMEM-F12, supplemented with 10% fetal bovine serum, 1% MEM nonessential amino acids, and 50 *μ*g/mL gentamicin. Cells were maintained at 37°C under 5% CO_2_/95% air.

### 2.4. MTT Assay

SH-SY5Y cells were seeded into 96-well culture plates at a density of 2 × 10^5^ cells/mL and allowed to attach. Twenty-four hours after seeding, the cells were differentiated with retinoic acid (10 *μ*M) for 6 days prior to treatment. The differentiated cells were then pretreated for 24 h with TQ or TQRF diluted in serum-free medium at concentrations of 0.03–100 *μ*g/mL. The treated cells were then challenged with 250 *μ*M H_2_O_2_ for 3 h [17]. MTT (3-[4,5-dimethylthiazol-2-yl]-2,5-diphenyl-tetrazolium bromide) (Sigma, St. Louis, MO, USA) was added to all wells and allowed to incubate in the dark at 37°C for 4 h. The amount of MTT formazan product was determined by measuring absorbance using a microplate reader (Opsys MR, Thermo Labsystems, Franklin, MA) at 570 nm.

### 2.5. Intracellular ROS Assay

Generation of ROS was evaluated using the 2′,7′-dichlorofluorescin diacetate (DCFH-DA) method. SH-SY5Y cells were seeded at a density of 2 × 10^5^ cells/mL in a black 96-well plate with a transparent bottom and allowed to attach. Twenty-four hours after seeding, the cells were differentiated with retinoic acid (10 *μ*M) for 6 days. Prior to treatment, cells were incubated with DCFH-DA (10 *μ*M) for 30 min in the dark at 37°C and washed twice with PBS. The cells were then pretreated with different concentrations of TQ or TQRF for 24 h and subsequently challenged with 250 *μ*M H_2_O_2_ and subjected to fluorescence measurement for 3 h using a microplate fluorescence reader (H1M, Bio-Tek Instrumentation, USA), at *λ*_exc_ = 480 nm and *λ*_em_ = 510 nm. The fluorescence intensity is proportional to the intracellular ROS levels.

### 2.6. Morphological Assessment by Inverted Light Microscope

SH-SY5Y cells were seeded into 6-well plates at a density of 2 × 10^5^ cells/mL and allowed to attach. Twenty-four hours after seeding, the cells were differentiated with retinoic acid (10 *μ*M) for 6 days prior to treatment. The cells were then pretreated with TQ or TQRF for 24 h and subsequently challenged with 250 *μ*M H_2_O_2_ for 3 h. The morphology of treated and untreated cells was observed by inverted light microscope (Nikon ECLIPSE TS100, Nikon Corporation, Tokyo, Japan) at 40x magnification and the images were captured using a digital camera (Nikon DS-Fi1, Nikon Corporation, Tokyo, Japan) and image acquisition software (NIS Elements D version 3.0). Multiple independent images were taken for each treatment.

### 2.7. Acridine Orange- (AO-) Propidium Iodide (PI) Staining

SH-SY5Y cells were seeded into 6-well plates at a density of 2 × 10^5^ cells/mL and allowed to attach. Twenty-four hours after seeding, the cells were differentiated with retinoic acid (10 *μ*M) for 6 days prior to treatment. The differentiated cells were then pretreated for 24 h with TQ or TQRF with subsequent exposure to 250 *μ*M H_2_O_2_ for 3 h. The cells were stained with the dye mixture of AO (50 *μ*g/mL) and PI (50 *μ*g/mL) and viewed under a confocal microscope (Olympus, Tokyo, Japan). Images were captured randomly (*n* = 6) at 20x magnification and the percentage of dead cells was determined; percentage of dead cells = (total number of apoptotic + necrotic cells/total number of cells counted) × 100. The stained orange and red indicated apoptotic and necrotic cells; meanwhile, the green nuclei indicated viable cells.

### 2.8. RNA Extraction

SH-SY5Y cells were seeded onto 6-well plates at density of 2 × 10^5^ cells/mL. The cells were differentiated with 10 *μ*M retinoic acid for 6 days prior to treatment. The cells were pretreated with 0.03 and 0.1 *μ*g/mL of TQ or TQRF for 24 h with subsequent exposure to 250 *μ*M H_2_O_2_ for 3 h. Total RNA was extracted using a Total RNA Isolation kit (RBC Bioscience Corp., Taiwan) according to the manufacturer's protocol. RNA concentration was quantified using a NanoDrop spectrophotometer (Thermo Scientific NanoDrop, NanoDrop Technologies, Wilmington, DE, USA), and the ratios of A260/230 and A260/280 were used to indicate the RNA purity.

### 2.9. Primer Design

Primers were designed from nucleotide sequences of the genes of interest and housekeeping genes obtained from the National Center for Biotechnology Information GenBank Database, while the internal control was supplied by Beckman Coulter Inc. (Miami, FL, USA) (Table 1). The specificity validation of the nucleotide sequences was performed using NCBI-nucleotide-BLAST. An additional 37 base pairs of universal tag sequences were attached to each forward and reverse primer. Synthesis of primers was performed by First Base Ltd. (Selangor, Malaysia) and diluted in 1x TE buffer to a concentration of 500 nM for reverse primers and 200 nM for forward primers [18].

### 2.10. cDNA Synthesis

The complementary DNA (cDNA) was synthesized from 50 ng/*μ*L RNA of each sample. The reverse transcription reaction (RT reaction) was performed according to the GenomeLab GeXP Start Kit instructions (Beckman Coulter Inc., Miami, FL, USA) using 1 *μ*L of each RNA sample, 4 *μ*L of 5x RT buffer, 2 *μ*L of RT multiplex reverse primers, 1 *μ*L of KanR, 1 *μ*L of reverse transcriptase, and 11 *μ*L of DNAse/RNase free water. cDNA was synthesized according to the reaction protocol: 48°C for 1 min, 42°C for 60 min, 95°C for 5 min, and 4°C hold in an XP Thermal Cycler (BIOER Technology, Hangzhou, China) [18].

### 2.11. PCR Amplification

PCR reactions were prepared using a GeXP Start Kit (Beckman Coulter, Brea, CA, USA) consisting of cDNA samples taken from the RT reaction (9.3 *μ*L each), 5x PCR buffer (2 *μ*L), 25 mM MgCl_2_ (2 *μ*L), PCR multiplex forward primer (1 *μ*L), and Thermo-Start DNA polymerase (0.7 *μ*L). Amplification conditions were 95°C for 10 min, followed by 34 cycles of 94°C for 30 sec, 55°C for 30 sec, 70°C for 1 min, and 4°C hold carried out in an XP Thermal Cycler (BIOER Technology, Hangzhou, China) [18].

### 2.12. GeXP Multiplex Data Analysis

Subsequently, the PCR product (1 *μ*L each) was mixed with 38.5 *μ*L of sample loading solution along with 0.5 *μ*L of DNA Size Standard-400 obtained from the GenomeLab GeXP Start Kit. The PCR products were then separated in the GenomeLab GeXP Genetic Analysis System (Beckman Coulter, Brea, CA, USA) by capillary gel electrophoresis according to their nucleotide sizes. The dye signal strength was measured in arbitrary units (AU) of optical fluorescence. The data were analyzed using the Fragment Analysis module of the GeXP system software and then transferred to the analysis module of the eXpress Profiler software. Normalization was performed with reference gene *β*-actin, according to manufacturer's instructions [18].

### 2.13. Statistical Analysis

Statistical analysis was conducted by one-way analysis of variance and Tukey's multiple comparison using Statistical Package for the Social Sciences (SPSS Inc., Chicago, Illinois, USA) version 21.0, and *p* < 0.05 was considered as significantly different.

## 3. Results

### 3.1. Protective Effects of TQRF and TQ on H_2_O_2_-Induced Neurotoxicity in SH-SY5Y Cells

**Figure 1:**
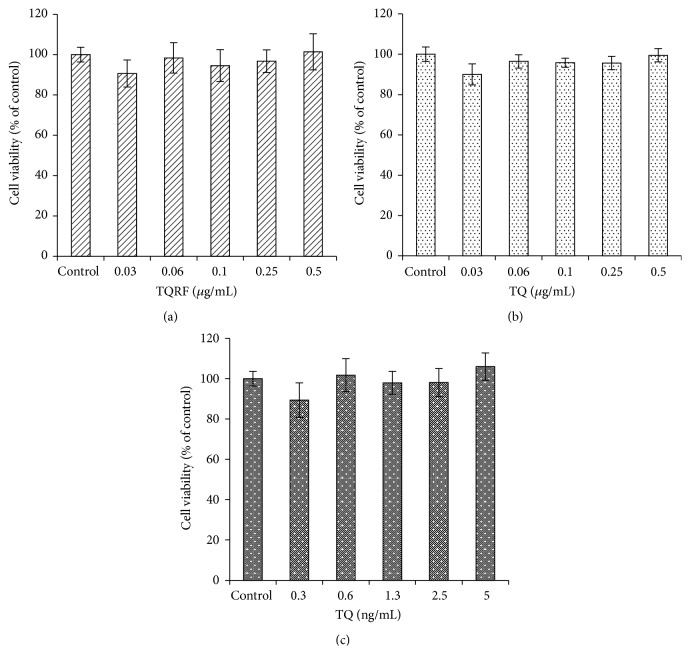
Viability (MTT assay) of SH-SY5Y cells treated with Thymoquinone-rich fraction (TQRF) and Thymoquinone (TQ) alone, respectively, for 24 h. (a) TQRF, (b) TQ (*μ*g/mL), and (c) 1% TQ in TQRF (ng/mL). Results are the mean ± SD. No significant difference between the control and treated groups.

**Figure 2:**
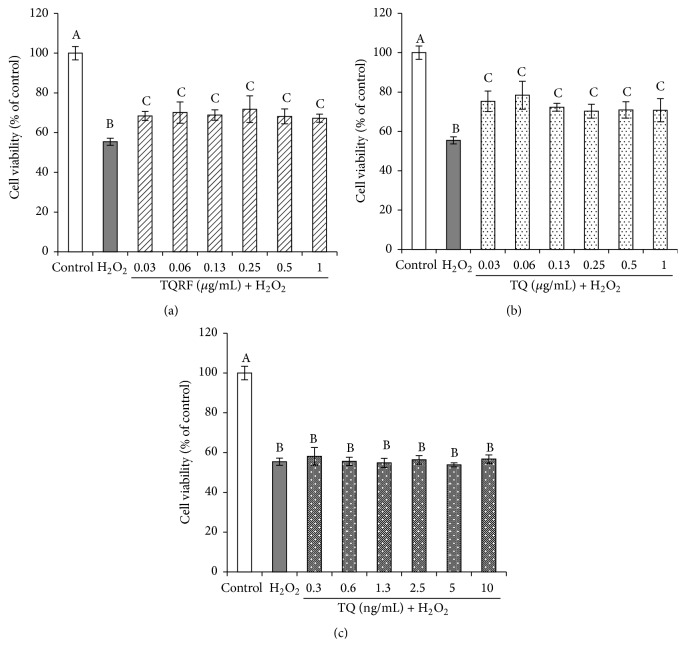
Viability (MTT assay) of SH-SY5Y cells pretreated with Thymoquinone-rich fraction (TQRF) and Thymoquinone (TQ) for 24 h and subsequent exposure to 250 *μ*M H_2_O_2_ for 3 h. (a) TQRF + 250 *μ*M H_2_O_2_, (b) TQ (*μ*g/mL) + 250 *μ*M H_2_O_2_, and (c) 1% TQ in TQRF (ng/mL) + 250 *μ*M H_2_O_2_. Results are the mean ± SD. In each panel, mean values labeled with different alphabets are significantly different at *p* < 0.05.

The cytotoxicity of TQRF and TQ on SH-SY5Y cells was initially determined in the absence of H_2_O_2_. As shown in Figure 1, the cell survival when TQRF and TQ were used to treat cells varied from 90 to 100%. The neuroprotective effects of TQRF and TQ were then determined by pretreating the cells with TQRF and TQ for 24 h and further exposure to 250 *μ*M of H_2_O_2_ for another 3 h. We have previously demonstrated that 250 *μ*M of H_2_O_2_ was able to induce approximately 50% cell death (IC_50_) [17], and therefore it was applied for our subsequent experiments. Moreover, in the present study, the cell survival when 250 *μ*M H_2_O_2_ was used alone was 56%, (*p* < 0.05) (Figures 2(a) and 2(b)). Additionally, cell survival for the TQRF- and TQ-pretreated cells (0.03–1 *μ*g/mL), followed by 250 *μ*M H_2_O_2_, was 60–70%, with 5–15% recovery of cell viability in comparison to H_2_O_2_ alone. TQ at a similar concentration that is present in TQRF (1%) (0.3–10 ng/mL) did not show any protective effect on 250 *μ*M of H_2_O_2_ (Figure 2(c)).

### 3.2. Effects of TQRF and TQ on H_2_O_2_-Induced ROS Production

**Figure 3:**
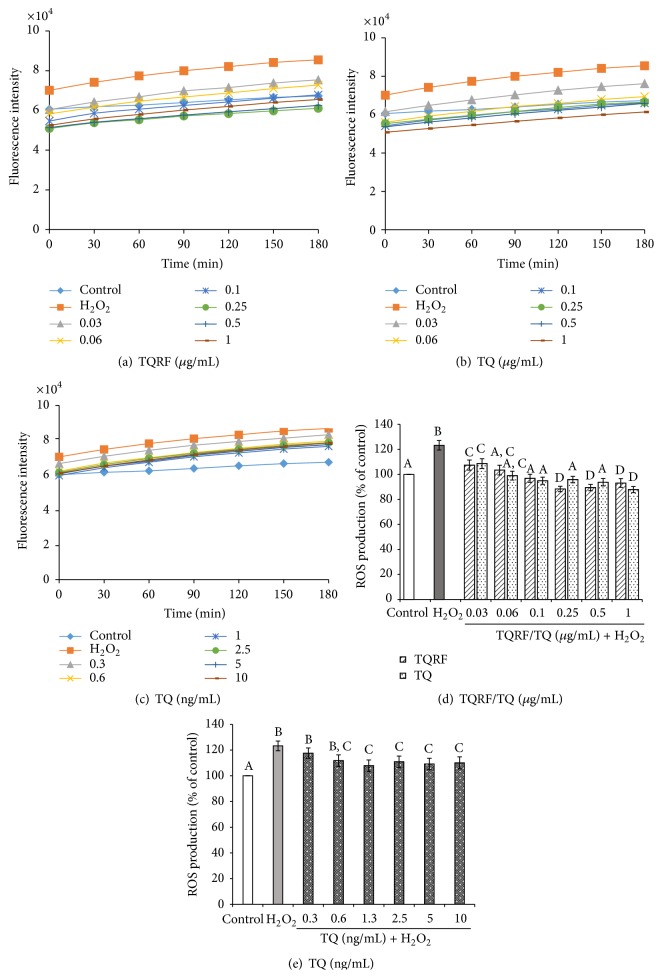
Effects of Thymoquinone-rich fraction (TQRF) and Thymoquinone (TQ) on intracellular ROS production. SH-SY5Y cells treated with different concentrations of (a) TQRF, (b) TQ (*μ*g/mL), and (c) 1% TQ in TQRF (ng/mL) prior to H_2_O_2_ (250 *μ*M) exposure. The fluorescence intensities for each treatment were recorded versus time up to 3 h. The percentage of ROS production (mean ± SD) relative to control cells in the presence of (d) TQRF/TQ (*μ*g/mL) and (e) TQ (ng/mL). In each panel, mean values labeled with different alphabets are significantly different at *p* < 0.05.

SH-SY5Y cells pretreated with similar concentrations of TQRF and TQ (0.03–1 *μ*g/mL) and subsequently incubated with H_2_O_2_ (Figure 3) significantly attenuated H_2_O_2_-induced generation of intracellular ROS (*p* < 0.05). However, 1% TQ (0.3–10 ng/mL) produced weaker effects. We thus concluded that the 1% TQ content in TQRF is not responsible for the TQRF-protective effects on H_2_O_2_-induced neurotoxicity and ROS generation in the cells. Subsequently, only TQRF and TQ at similar concentrations (0.03–1 *μ*g/mL) were compared.

### 3.3. Morphological Assessment by Inverted Light Microscope

**Figure 4:**
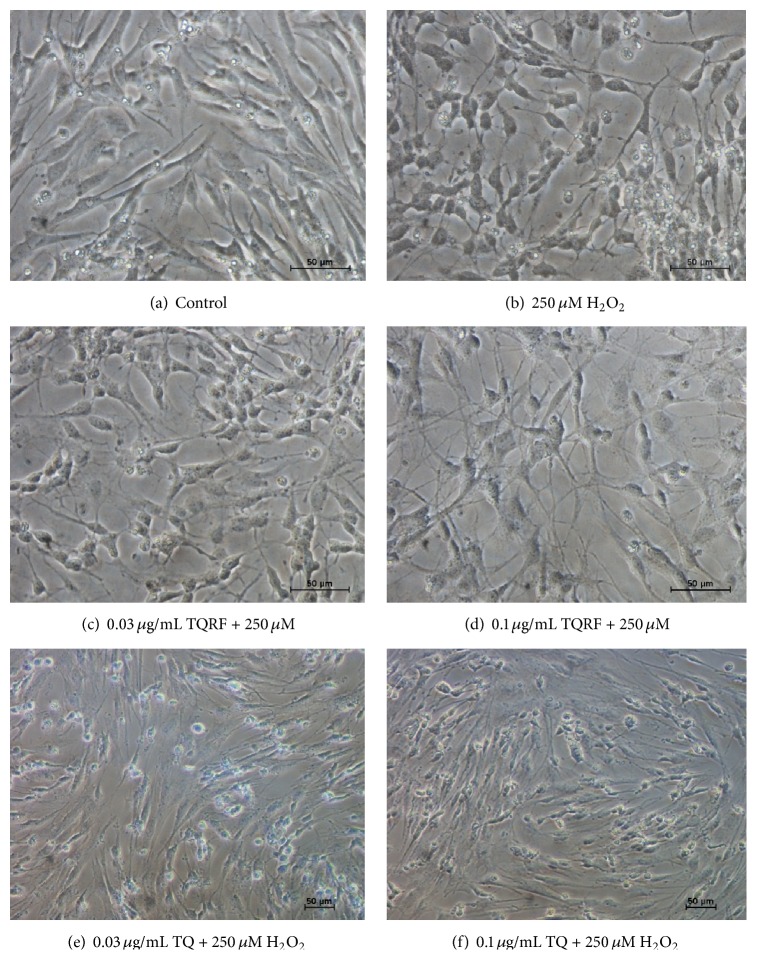
Phase-contrast micrograph observation on SH-SY5Y at 40x magnification. (a) Untreated cells (control), (b) 250 *μ*M H_2_O_2_ alone, (c) 0.03 *μ*g/mL TQRF + 250 *μ*M H_2_O_2_, (d) 0.1 *μ*g/mL TQRF + 250 *μ*M H_2_O_2_, (e) 0.03 *μ*g/mL TQ + 250 *μ*M H_2_O_2_, and (f) 0.1 *μ*g/mL TQ + 250 *μ*M H_2_O_2_.

Morphological assessment of the cells revealed H_2_O_2_-induced features of cell damage such as cell loss, neuritis retraction, and cell shrinkage (Figure 4(b)) in comparison with the control cells (Figure 4(a)). Pretreatment with TQRF and TQ at 0.03 and 0.1 *μ*g/mL exerted a protective effect on H_2_O_2_-induced neurotoxicity as the cell integrity remained preserved (Figures 4(c)–4(f)).

### 3.4. AO-PI Staining of SH-SY5Y Cells

**Figure 5:**
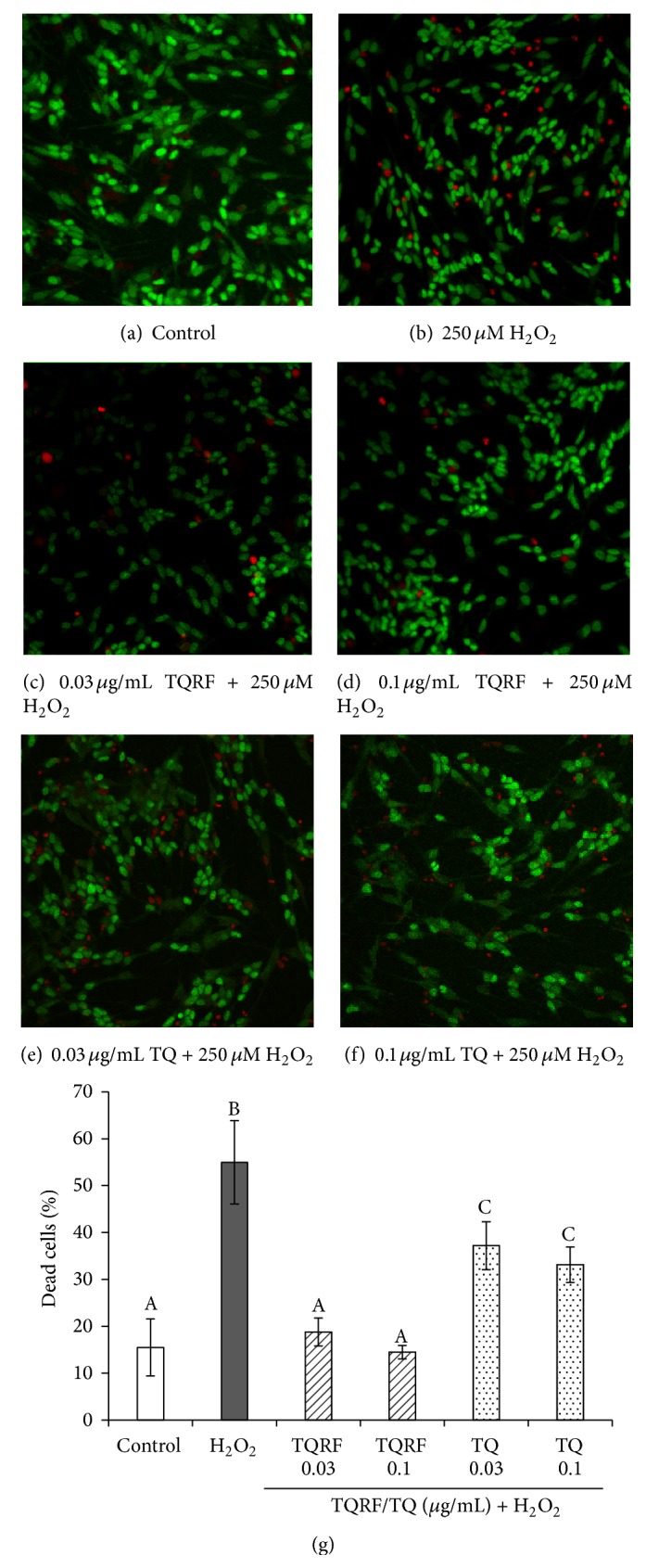
Acridine orange- (AO-) propidium iodide (PI) double staining cell morphological assessment. Morphological changes of SH-SY5Y cells pretreated with Thymoquinone (TQ) and Thymoquinone-rich fraction (TQRF) for 24 h followed by subsequent exposure to 250 *μ*M H_2_O_2_ for 3 h. (a) Untreated cells (control), (b) 250 *μ*M H_2_O_2_ alone, (c) 0.03 *μ*g/mL TQRF + 250 *μ*M H_2_O_2_, (d) 0.1 *μ*g/mL TQRF + 250 *μ*M H_2_O_2_, (e) 0.03 *μ*g/mL TQ + 250 *μ*M H_2_O_2_, and (f) 0.1 *μ*g/mL TQ + 250 *μ*M H_2_O_2_. Viable cells are stained green by acridine orange, while late apoptotic and necrotic cells are stained orange and red by propidium iodide. (g) The percentage of dead cells. Values represent mean ± SD. Mean values labeled with different alphabets are significantly different at *p* < 0.05.

AO-PI double staining distinguishes viable, apoptotic, and necrotic cells. The untreated control cells showed round and green nuclei, which indicated viable cells (Figure 5(a)). Late apoptotic and necrotic cells were stained orange and red as displayed in H_2_O_2_ alone (Figure 5(b)). Pretreated cells (TQRF and TQ) showed fewer orange- and red-stained nuclei in comparison with H_2_O_2_ alone (Figures 5(c)-5(d) and 5(e)-5(f)). The percentage of dead cells was presented in a bar graph (Figure 5(g)). H_2_O_2_ induced toxicity to the cells as significant dead cells were noticed in H_2_O_2_ alone in comparison to untreated cells (control). In the pretreated groups, the images of the neuroprotective effect of 0.03 *μ*g/mL of TQRF or TQ were not as evident as 0.1 *μ*g/mL; however, they were not significantly different (Figure 5(g)).

### 3.5. Nutrigenomic Modulation of Antioxidant and Apoptotic Genes by TQRF and TQ

**Figure 6:**
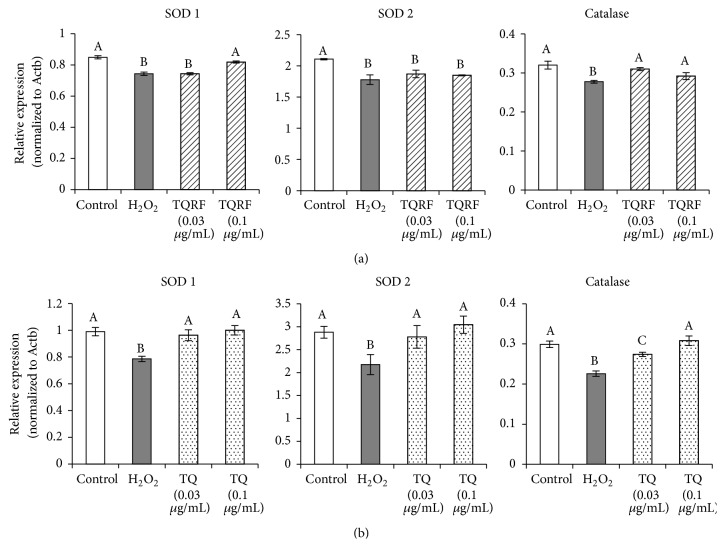
GeXP multiplex gene expression analysis of antioxidant genes (SOD1, SOD2, and catalase). Pretreatment with (a) Thymoquinone-rich fraction (TQRF) and (b) Thymoquinone, and subsequent exposure to 250 *μ*M H_2_O_2_. Results are the mean ± SD. In each panel, mean values labeled with different alphabets are significantly different at *p* < 0.05.

H_2_O_2_ insult on SH-SY5Y cells downregulated antioxidant genes SOD1, SOD2, and catalase, in contrast with the untreated cells (Figures 6(a) and 6(b)). Pretreatment of cells with TQRF at 0.1 *μ*g/mL prior to H_2_O_2_ exposure upregulated SOD1 and catalase genes, in comparison with H_2_O_2_ alone. However, no difference was observed for SOD2 expression between H_2_O_2_- and TQRF-treated groups (Figure 6(a)). Meanwhile, cells pretreated with TQ (0.03 and 0.1 *μ*g/mL) prior to H_2_O_2_ exposure upregulated SOD1, SOD2, and catalase genes in comparison with those treated with H_2_O_2_ alone (*p* < 0.05) (Figure 6(b)).

**Figure 7:**
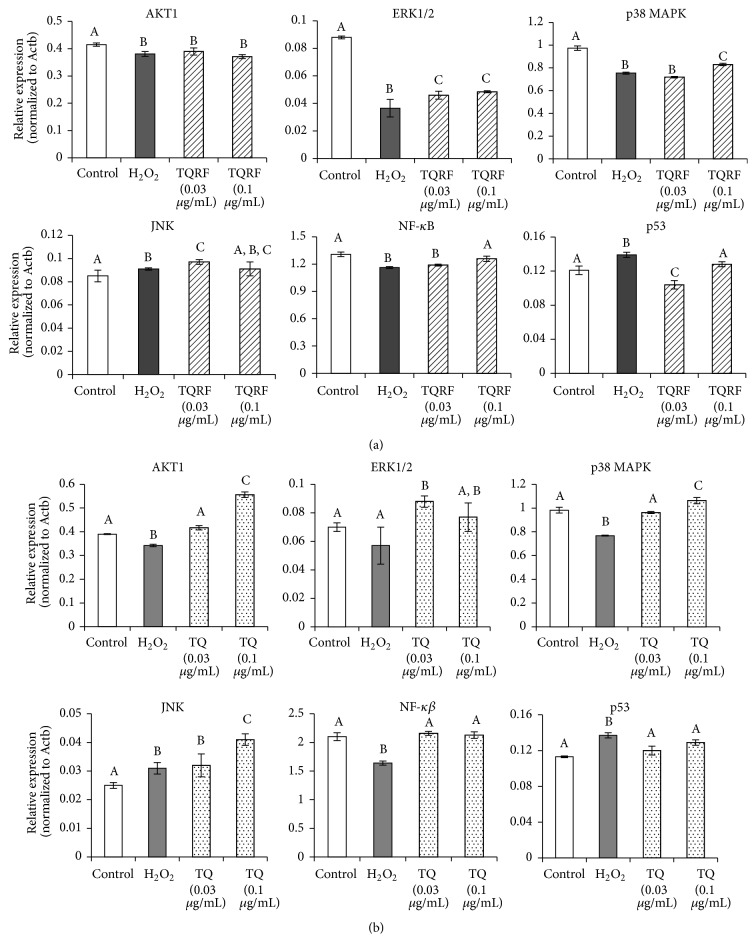
GeXP multiplex gene expression analysis of apoptotic genes (AKT1, ERK1/2, p38 MAPK, JNK, NF-*κβ*, and p53). Pretreatment with (a) Thymoquinone-rich fraction (TQRF) and (b) Thymoquinone (TQ), and subsequent exposure to 250 *μ*M H_2_O_2_. Results are the mean ± SD. In each panel, mean values labeled with different alphabets are significantly different at *p* < 0.05.

On the other hand, cells exposed to H_2_O_2_ alone upregulated JNK and tumor protein p53 (p53) gene expression levels and downregulated AKT1, ERK1/2, p38 MAPK, and NF-*κβ*, in comparison with the untreated controls (Figures 7(a) and 7(b)). Pretreatment of cells with TQRF prior to H_2_O_2_ insult downregulated p53 gene expression level and upregulated the expressions of ERK1/2, p38 MAPK, and NF-*κβ* genes (*p* < 0.05). No changes were noticed in AKT1 and JNK between H_2_O_2_ alone and the TQRF-treated groups (Figure 7(a)). Pretreatment of cells with TQ prior to H_2_O_2_ insult also downregulated the p53 gene expression level and upregulated the expression of AKT1, ERK1/2, p38 MAPK, JNK, and NF-*κβ* genes (*p* < 0.05) (Figure 7(b)).

## 4. Discussion

The neuroprotective effects of TQ have partly been reported. TQ attenuated A*β*_25–35_-induced toxicity on differentiated rat PC12 cells [19] and protected the rat cerebellar granule neurons against neurotoxin A*β*_1–40_ [20, 21]. Nevertheless, information on the mechanism of action of TQ and the beneficial effects of TQRF has been scarce. In this study, we demonstrated that TQRF protected human neuronal cells from H_2_O_2_-induced cell damage over and above that produced by an equal amount of TQ present in TQRF. This is evidenced by the neuroprotective effects of TQRF in comparison to 1% TQ against H_2_O_2_, as determined by MTT assay (Figure 2) and ROS generation (Figure 3). Subsequently, TQRF and TQ at similar concentrations were compared. Both TQRF and TQ pretreatment showed their neuroprotective properties partly through attenuation of morphological features of apoptosis and cell damage when exposed to H_2_O_2_, observed under light microscopic and fluorescent staining with AO and PI dyes (Figures 4 and 5).

Furthermore, much evidence showed that H_2_O_2_ induced apoptosis [22, 23] and redox changes [24] when exposed to the cells. Underlying changes include activation of antioxidant defense systems in neuronal cells to counter H_2_O_2_, which may overwhelm the cells if in excess. If the level of ROS exceeds the protection of endogenous antioxidant systems, cell death is likely and is implicated in neurodegeneration [25]. In the presence of exogenous antioxidants like TQRF and TQ, the endogenous antioxidant system is potentiated, as suggested by the present data in which TQRF and TQ (Figure 6) increased the expression of antioxidant genes to further protect the cells. In the present study, the effect of TQRF and TQ on antioxidant expression suggests that they may potently prevent the redox changes that favor neurodegeneration.

Besides the antioxidant defense mechanism, activation of Akt and MAPK pathways also plays major roles in cell growth, survival, differentiation, and apoptosis responses [26]. In SH-SY5Y cells, the Akt pathway elicits survival signaling following various stresses, and this signaling leads to the inhibition of apoptosis [27]. Notably, following oxidative injury, AKT is responsible for the preventive effects on neural cell apoptosis [28]. In the present study, TQRF and TQ pretreatment increased AKT expression. Also, oxidative stress is one of the major stimuli for MAPK cascades which are involved in apoptotic signal transduction. H_2_O_2_ induces oxidative damage to neuronal cells through modulation of apoptotic mechanisms involving the activation of MAPKs [29]. H_2_O_2_ exposure downregulated ERK1/2 gene expression; however, that expression was upregulated with TQRF and TQ pretreatment. ERK activation is implicated as a survival factor following oxidative injury [30, 31]. Thus, in response to H_2_O_2_-induced oxidative stress, TQRF and TQ pretreatment may have activated ERK1/2 gene expression as one of its survival mechanisms of action following oxidant insult.

In addition, p38 MAPK is a stress kinase, which is activated by stimuli capable of causing cell death. As a consequence, p38 MAPK is generally considered as a cell death mediator. Its induction by H_2_O_2_ in the present study likely activated JNK [32], which is known to be involved in proapoptotic signaling [33]. JNK activation facilitates the decrease of mitochondrial membrane potential followed by release of cytochrome c which then activates caspase-9 and caspase-3, eventually leading to cell death [34]. Our results showed that H_2_O_2_-induced JNK activation was attenuated by TQRF and TQ treatment, suggesting that the protective effects of TQRF and TQ on H_2_O_2_-induced injury in SH-SY5Y cells also involved mitochondrial protection. Furthermore, TQRF and TQ pretreatment enhanced the gene expression of NF-*κ*B, indicating that the NF-*κ*B signaling pathway is one of its survival and antiapoptotic mechanisms of action in response to oxidative insult. On the other hand, the tumor suppressor protein p53 is a key regulator of cell cycle, senescence, or apoptosis. p53 induces the expression of target genes and causes cell-cycle arrest, DNA repair, or apoptosis in response to cellular genotoxic stresses [35, 36]. The present study showed that H_2_O_2_-induced cell damage likely affected the cellular genotoxic stress involving p53. However, pretreatment with TQRF and TQ reduced the p53 gene expression level, indicating that TQRF and TQ may protect the neuronal cells against ROS insult through modulating p53. By and large, neurodegenerative diseases are associated with ROS generation, the attenuation of which has been demonstrated to be effective in preventing oxidative cell damage [37].

**Figure 8:**
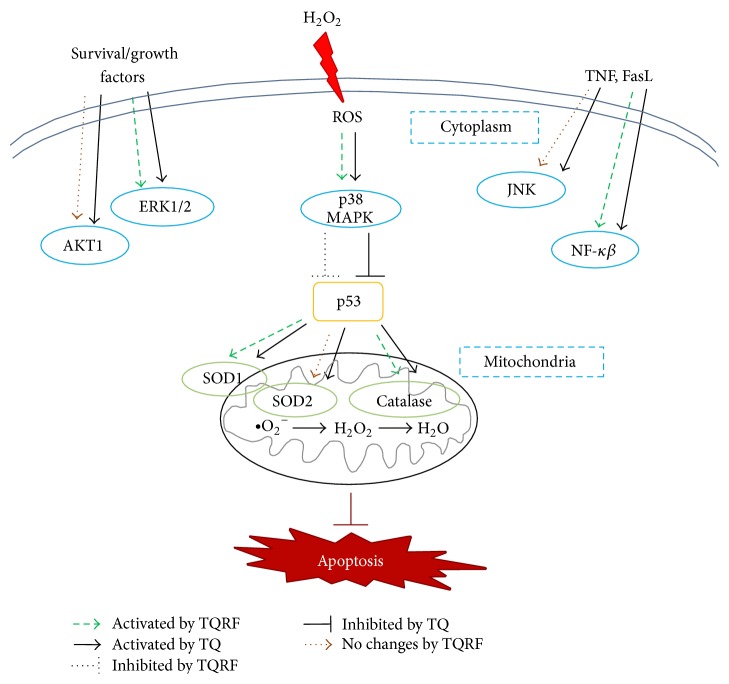
Schematic presentation of the proposed mechanistic basis for the neuroprotective effects of TQRF and TQ against H_2_O_2_-induced neurotoxicity in human differentiated SH-SY5Y cells at mRNA transcriptomics level. Oxidative stress modulated the cellular redox status and activated proapoptotic genes (i.e., JNK and p53) and downregulates antiapoptotic genes (i.e., AKT1, ERK1/2, p38 MAPK, and NF-*κβ*), resulting in cellular apoptosis. In contrast, TQRF and TQ upregulated endogenous antioxidant defences, while they downregulated proapoptotic genes and activated antiapoptotic genes leading to enhanced cell survival.

In the present study, the results demonstrated that TQRF protected cells from H_2_O_2_-induced toxicity via multiple transcriptional mechanisms (Figure 8) better than TQ alone. This can be attributed to the presence of other bioactive compounds in TQRF that may have synergistically improved the activity of TQ. This is supported by Imam et al. [12], who reported on the efficacy of bioactive compounds in comparison with their rich fractions; multiple compounds in an extract produced better activity than single compounds. This concept of rich fractions underscores the importance of using rich extracts instead of refined or synthetic compounds in pharmacological studies. Indeed, this property has been noted before with a number of spices [38].

Nowadays, diet is no longer simple nutrition since the evolvement of nutrigenomics, forging the impact of the foods we eat on the function of how our genes respond as seen in the modulation of apoptosis and cell survival against oxidative insult. In future, a nutrigenomic approach may allow for the elucidation of genomic and cellular regulating mechanisms, particularly their role in the deterioration of normal healthy processes and in the initiation of disease processes. In the study of neurodegenerative diseases, nutrigenomics augments the discovery of neuroprotective pathways using diet and through the use of new natural substances that may induce the expression of health-promoting genes and reduce the disease-promoting genes [39].

## 5. Conclusion

The ability of dietary antioxidants to potentially prevent or at least retard the progression of neurodegenerative diseases has been linked to their ability to prevent neuronal death and enhance cell survival through prevention of oxidative stress-induced apoptosis. In aggregate, we have demonstrated that TQRF extracted from* N. sativa* using a green technology SFE system was more effective than TQ alone, when an equal concentration of TQ in TQRF was compared with H_2_O_2_-induced neurotoxicity on human neuronal SH-SY5Y cells. Additionally, TQRF showed comparable effects to TQ when the same concentrations were tested. The results suggest that TQRF and TQ are potential candidates for the prevention of neurodegenerative disease due to oxidative injury. The present study proposes the nutrigenomic basis of the neuroprotective effects exhibited by TQRF and TQ at the transcriptomic level of antioxidants and apoptotic signaling genes. The study on gene expression using multiplex GeXP provides some insight into and early indicators on the mechanisms involved. Further study concerning the protein expression should be carried out together with the use of selective inhibitors of the proposed pathways. Furthermore, an animal study as the whole organism would be appropriate to ascertain the schematic mechanism of the actions proposed.

## Figures and Tables

**Table 1:** Gene name, accession number, and reverse and forward primer sequences used in GeXP multiplex gene expression analysis.

Gene name	Accession number	Primer sequences^*∗*^ with universal tags	
		Forward	Reverse
Antioxidant genes			
SOD 1	NM_000454	AGGTGACACTATAGAATATCATCAATTTCGAGCAGAAGG	GTACGACTCACTATAGGGATGCTTTTTCATGGACCACC
SOD 2	NM_000636	AGGTGACACTATAGAATACATCAAACGTGACTTTGGTTC	GTACGACTCACTATAGGGACTCAGCATAACGATCGTGGTT
Catalase	NM_001752	AGGTGACACTATAGAATAGAAGTGCGGAGATTCAACACT	GTACGACTCACTATAGGGAACACGGATGAACGCTAAGCT

Apoptotic genes			
ERK1/2	NM_002745	AGGTGACACTATAGAATAGGAGCAGTATTACGACCCGA	GTACGACTCACTATAGGGAGATGTCTGAGCACGTCCAGT
p53	NM_001126117	AGGTGACACTATAGAATAGGGGAGCAGGGCTCA	GTACGACTCACTATAGGGAAAAATGGCAGGGGAGGG
JNK	NM_139046	AGGTGACACTATAGAATACAGAAGCTCCACCACCAAAGAT	GTACGACTCACTATAGGGAGCCATTGATCACTGCTGCAC
AKT1	NM_001014431	AGGTGACACTATAGAATAGAGGAGATGGACTTCCGGTC	GTACGACTCACTATAGGGAAGGATCTTCATGGCGTAGTAGC
NF-kB	NM_001077493	AGGTGACACTATAGAATAGCGGGCGTCTAAAATTCTG	GTACGACTCACTATAGGGATTCCACGATCACCAGGTAGG
p38	NM_001315	AGGTGACACTATAGAATATTCAGTCTTTGACTCAGATGCC	GTACGACTCACTATAGGGAGTCAGGCTTTTCCACTCATCT

Housekeeping genes			
GAPDH^a^	NM_002046	AGGTGACACTATAGAATAAAGGTGAAGGTCGGAGTCAA	GTACGACTCACTATAGGGAGATCTCGCTCCTGGAAGATG
Hyaluronidase^a^	AJ000099	AGGTGACACTATAGAATACAGCAGTTCATGCTGGAGAC	GTACGACTCACTATAGGGACCAGGTAGACAGACGGGAAG
18 sRNA^a^	M10098	AGGTGACACTATAGAATAGGAGTGGAGCCTGCGGCTTAA	GTACGACTCACTATAGGGATAGCATGCCAGAGTCTCGTT
Actb^a,#^	NM_001101	AGGTGACACTATAGAATAGATCATTGCTCCTCCTGAGC	GTACGACTCACTATAGGGAAAAGCCATGCCAATCTCATC
Kan(r)^b^	—	AGGTGACACTATAGAATAATCATCAGCATTGCATTCGATTCCTGTTTG	GTACGACTCACTATAGGGAATTCCGACTCGTCCAACATC

^*∗*^Based on the *Homo sapiens* gene sequences adopted from the National Center for Biotechnology Information GenBank Database.

^a^Housekeeping genes, ^b^Internal control, and ^#^Normalization gene.

## Abbreviations

*N. sativa*: * Nigella sativa* Linn.
TQ: Thymoquinone
TQRF: Thymoquinone-rich fraction
H_2_O_2_: Hydrogen peroxide
ROS: Reactive oxygen species
SFE: Supercritical fluid carbon dioxide extraction
DMEM-F12: Dulbecco's minimum essential Eagle's medium-Ham's nutrient mixture F-12
PBS: Phosphate-buffer saline
MgCl_2_: Magnesium chloride
MTT: 3-[4,5-Dimethylthiazol-2-yl]-2,5-diphenyl-tetrazolium bromide
DCFH-DA: 2′,7′ Dichlorofluorescein diacetate
AO: Acridine orange
PI: Propidium iodide
cDNA: Complementary DNA
RT: Reverse transcription
SOD: Superoxide dismutase
JNK: c-Jun N-terminal kinase
p53: Tumor protein p53.
